# Prevalence of frailty and contributory factors in three Chinese populations with different socioeconomic and healthcare characteristics

**DOI:** 10.1186/s12877-015-0160-7

**Published:** 2015-12-09

**Authors:** Jean Woo, Zheng Zheng, Jason Leung, Piu Chan

**Affiliations:** Department of Medicine & Therapeutics, The Chinese University of Hong Kong, 9/F, Lui Che Woo Clinical Sciences Building, Prince of Wales Hospital, Shatin, N. T Hong Kong; Beijing Institute of Geriatrics, Beijing, China; The Jockey Club Centre for Osteoporosis Care and Control, The Chinese University of Hong Kong, Hong Kong, Hong Kong

**Keywords:** Frailty, Compression of morbidity, Polypharmacy, Multi-morbidity

## Abstract

**Background:**

Frailty predicts dependence and mortality, and is an important health indicator for aging populations. Comparing frailty prevalence between populations of the same ethnicity but different socioeconomic, lifestyle, health and social care systems, and environmental characteristics would address the role of these factors in contributing to frailty.

**Methods:**

We compare frailty prevalence and contributory factors across three Chinese populations: Beijing rural, Beijing urban, and Hong Kong (urban). Older people aged 65 years and above living in the community were invited to respond to a general health questionnaire covering demographic, socioeconomic, medical and drug histories, geriatric syndromes, assessment of physical and cognitive functioning, psychological wellbeing and nutritional status. Frailty is defined as an index calculated from multiple deficits > = 0.25 (FI). The ratio of FI/life expectancy at birth was used as an indicator of compression of morbidity. Risk factors and attributable fraction for frailty were compared across the three cohorts.

**Results:**

The prevalence of frailty increases with age in all three cohorts, and was lower among rural compared with urban (Beijing and Hong Kong) populations. The highest FI/LE ratio was observed in the Beijing urban population, followed by Hong Kong, with the Beijing rural population having the lowest ratio. Risk factors for frailty were similar in all three populations. Those having the highest ORs were multi-morbidity (number of diseases > = 3), polypharmacy (number of drugs > = 4), age 85+, female gender, followed by low education level, and physical inactivity. For all three cohorts, age and multi-morbidity constitute the highest attributable fraction, and were highest in the Beijing rural cohort. A major difference between the Beijing and Hong Kong cohorts is the high AF from polypharmacy in Beijing and the ‘protective’ contribution of being married; and the effect of being a teetotaler in the Hong Kong cohort.

**Conclusions:**

This comparison draws attention to the importance of frailty prevention for ageing populations.

## Background

Population ageing is a world-wide phenomenon, for developing as well as developed countries. Chinese men and women living in Hong Kong (a Special Administrative Region of China) have the longest life expectancy in the world second only to Japan. The population in mainland China is also ageing rapidly, the increase in life expectancy being higher in urban compared with rural areas. With such demographic changes public health attention has been directed towards non communicable diseases and disability. Although increasing life expectancy is a result of improving socioeconomic status as well as healthcare, there is a suggestion in many countries that life expectancy increase is accompanied by increasing multi-morbidity and disability [1]. The latter are preceded by frailty, a state representing decline in functional reserves (both physical and cognitive), and results in adverse outcomes and ‘societal burden’. Frailty is a geriatric syndrome that is manifested as a result of multisystem impairments that predisposes to disability, morbidity, dependence and institutionalization [2, 3]. Frailty may be assessed using the phenotype approach consisting of five items, while a second approach counts the number of things that people have wrong with them rather than specific items, to calculate and index that represents cumulative deficits [4]. An ideal goal would be increase in life expectancy without increasing frailty. Therefore frailty may be considered a pertinent public health indicator for how well populations are aging, in addition to the more traditional indicators such as multi-morbidity and disability. A recent survey of frailty prevalence in low- and middle-income countries as part of the 10/66 population-based cohort study documented frailty as a relevant construct that predicts dependence and mortality [5].

There are few studies comparing frailty prevalence between populations. Such between population studies would enable examination of the role of personal, environmental, and health/social care systems in contributing to frailty, although it is known that they all contribute to life expectancy. Comparison across populations with differing characteristics, but the same ethnicity, would allow these contributors to be defined independent of any ethnic differences in predisposition to frailty. In this study we compare frailty prevalence and contributory factors across three Chinese populations: Beijing rural, Beijing urban, and Hong Kong (urban). There are environmental differences between Beijing and Hong Kong, most notably in air pollution, climate, food and water quality. The health and social care systems between Beijing and Hong Kong also differ, the key being an essentially free secondary/tertiary health care system and well developed social services in Hong Kong financed by taxation. However both regions have poorly developed primary care which largely relies on out of pocket expenses.

### Participants

In Beijing, 10,039 community-living participants were recruited in Beijing Longitudinal Study of Aging II (BLSA II) project [6] from July to November 2009, using a multistage cluster random sample of Beijing residents older than age 55 years from three urban and one rural district. Ethical approval was obtained from the Research Ethics Committee of Xuanwu Hospital of the Capital Medical University. Subjects over the age of 65 years (*n* = 7298) are included in this analysis. In Hong Kong, 4000 participants were recruited by placing advertisements in housing estates and community centres, as part of a bone health survey. Mr, Os and Ms. Os (Hong Kong) study between August 2001 and December 2003 [7, 8]. The sample was stratified to recruit approximately the same numbers of people in each of the three age strata: 65–74, 75–84, 85+ [9]. Ethical approval was obtained from the Clinical Research Ethics Committee of the Chinese University of Hong Kong and Hospital Authority NTE Cluster. Written informed consents were obtained from all participants in both sites.

## Methods

For both sites, information from questionnaire and clinical measurements were collected. These include demographic, socioeconomic, history of chronic diseases, drugs, geriatric syndromes, functional assessment, psychological well being, cognitive function, nutritional status, and lifestyle habits. Blood tests were also carried out in the Beijing cohort. The frailty index [FI] was constructed from 34 items [Table 1]. The FI is calculated as the number of items that represent a deficit divided by the total number of items. A cut point of > = 0.25 was used to indicate frailty [4]. Previous studies in China and Hong Kong have examined frailty prevalence using the FI, and shown that frailty is associated with increased use of hospital services and mortality [10–12], and influenced by social determinants [13]. In order to examine factors that may contribute to frailty that are common to both datasets, we selected items relating to chronic diseases and geriatric syndromes, medication, and lifestyle variables, as these have been shown to be associated with frailty [13–15].

**Table 1:** Comparison of Frailty index

Type	Questions	Item in Beijing	Item in Hong Kong^a^
Chronic disease history^b^	Hypertension	1	1
	Cardiovascular disease	2	2
	COPD	3	3
	Stroke	4	4
	Dementia	5	5
	Diabetes type I or II	6	6
	Arthritis	7	7
	Tumor	8	8
	Cataract	9	9
	Heart failure	10	10
	Kidney failure	11	11
	Deaf	12	-
	Thyroid disease	-	12
Functional assessment	Geriatric Depression Score ≥ 8	13	13
	MNA < 24	14	14
	MMSE < 24	15	15
	Tinetti’s Mobility Test (POMA) < 24	16	-
	Repeated chair stand (5 stands) > 15 sec	-	16
Geriatric syndromes	Joint pain or inflammation	17	17
	Gout	18	18
	Risk of fall^c^	19	19
	Osteoporosis	20	20
	Arterial Sclerosis	21	21
	Difficulty in movement^d^	22	22
	Less activity	23	23
	Often feel fatigue or tired	24	24
	Weight loss > 3 kg in past 3 months	25	25
	Vision loss in past 3 months	26	26
	Urinary inconsistence	27	-
	Fecal inconsistence	28	-
	Memory loss	29	-
	Hearing loss in past 3 months	30	-
	Difficulty in climbing several stairs	-	27
	Accomplished less daily activities due to emotional problem	-	28
	Difficulty in moderate activities	-	29
	Difficulty in social life	-	30
	Fall in past 12 months	-	31
	Poor health	-	32
	Ever fracture	-	33
Physical or lab examination	BMI < 19	31	34
	Dsylipideamia (mmol/l)^e^	32	-
	Plasma fasting glucose (mmol/l)^f^	33	-
	Blood urine acid^g^	34	-

^a^For 34 items in Beijing, there are 23 same items in Hong Kong

^b^Chronic disease history, “Did any doctor or clinic has diagnosed you have following diseases?”

^c^Risk of fall: any 2 or more of the following 6 questions: Q1. Do need a lot of efforts to reach objects above head? Q2 .Do you often wear large slippers or lose sleeping grown at home? Q3. Do you take a lot of efforts to pick up objects on the floor? Q4. Do you take a lot of efforts to step in or get out of bathtub? Q5.Do you take a lot of efforts to stand up from a chair or sit down? Q6. Do you need assistant of holding any thing while walking?

^d^Difficulty in movement: any 3 or more of the following 9 questions: Q1: Do you have smaller and smaller letters of your hand writing? Q2: Do you have weaker voice when you speak than before? Q3: Do you have less facial expression than before? Q4: Are you feeling rigidity or stiffness when you move? Q5: Do you have less waving hands or arms when walk? Q6: Is your hand or any part of body shaking sometimes? Q7: Do you often been ask for repeat because your voice was softer or unclear? Q8: Have you fell down in past 12 months? Q9: Do you lean forward with shuffling steps while walking?

^e^TG ≥ 200 or TCH ≥ 240 or LDL ≥ 160 or HDL < 40

^f^PFG > 110

^g^UA > 420 for male, UA > 360 for female

FI = x/34

While there may appear to be duplication of items in Table 1, such as dementia and memory loss, deafness and hearing loss etc., they represent deficits from different perspectives, since diagnosis of chronic diseases by doctors may differ from the list of geriatric syndromes. For example memory deficit may not equate with a diagnosis of dementia, while hearing loss may be due to wax in the external auditory meatus rather than a medical diagnosis of deafness. We also examined the construct of multi morbidity, by using the variable of the total number of chronic diseases (listed in Table 1) that are greater than or equal to three. This construct is increasingly important for aging populations worldwide, but may be distinguished from dependency and frailty by their different impact on health outcomes [16].

We hypothesized that the ratio of FI to life expectancy at birth (FI/LE) may be used as indicator of compression of morbidity for comparison between the three cohorts, lower values representing compression of morbidity with low FI and high LE. The life expectancy in 2014 for China is 73.1 for men and 77.4 for women [17]; for Hong Kong is 81.2 for men and 86.7 for women [18]. In both sites, interviews and measurements were done by trained research staffs. Study quality was supervised by study coordinators. Data inconsistency and missing values were queried and resolved before the study close-out. All items for FI had less than 5 % missing values and were dichotomized into the presence or absence of a frailty marker.

Beijing rural and Hong Kong were standardized by age (5-year groups) to that of the Beijing urban population. Population characteristics of Beijing urban, Beijing rural and Hong Kong were compared using analysis of variance (ANOVA) for continuous variables or Chi square test for categorical variables. Analyses were repeated by stratifying age 65–74, 75–84 and 85 or above. Prevalence of frailty was analyzed by Chi square test. Difference of FI/LE were examined by ANOVA. Risk factors were compared between the three cohorts using crude Odds ratios (OR). Variables with *P* < 0.1 are included into multiple logistic regression with backward variable selection method. The attributable fractions (AF) for risk factors contributing to frailty were then compared. The AF was calculated using the formula

AF = (OR-1)/OR. T tests were used for comparing mean continuous variables, log odds ratio of frailty and AUC in logistic regression. Chi square tests were used for comparing categorical variables. Statistical analyses were performed using the statistical package SAS, version 9.2 (SAS Institute, Inc., Cary, NC, USA). All statistical tests were two-sided. A *P*-value of <0.05 was considered statistically significant.

## Results

The characteristics for the three populations (Beijing urban, Beijing rural, and Hong Kong are compared in Table 2. There are differences in socioeconomic status, lifestyle, co morbidity, use of drugs, depressive symptoms and cognitive function between the three groups. Compared with the Beijing urban population, both the Beijing rural and Hong Kong men have lower education level, consume fewer drugs, have less depressive symptoms, and higher prevalence of cognitive impairment. The highest prevalence of being married occurred in Beijing urban men, followed by Hong Kong, and then Beijing rural men. Current smoking habit was commonest among Beijing rural men, followed by Beijing urban men, with the lowest prevalence among men in Hong Kong. More Hong Kong compared with Beijing men are married. The prevalence of current alcohol use was highest among Beijing rural men, while the prevalence for Beijing urban and Hong Kong were similar. Beijing urban and Hong Kong men were less active and had greater number of chronic diseases compared with Beijing rural men. A similar pattern was observed for women, with the following exceptions: Hong Kong women had the lowest prevalence of being currently married, the highest prevalence of living alone, and the highest prevalence of inactivity. These differences became less marked with successively older age groups (Tables 3, 4 and 5).

**Table 2:** Population characteristics between Beijing urban, Beijing rural and Hong Kong

	Mean (sd)/Freq (%)		
	Beijing urban (1)	Beijing rural (2)^a^	Hong Kong (3)^a^
Male	*N* = 2432	*N* = 419	*N* = 2000
Age, mean (sd)	74.62 (5.62)	74.89 (5.79)	74.47 (5.50)
Currently married	2136 (87.83 %)	365 (79.39 %)^1^	1760 (85.46 %)^1,2^
Education ≤ Middle school	632 (26.02 %)	248 (72.18 %)^1^	1422 (72.74 %)^1^
Living alone	149 (6.13 %)	19 (6.80 %)	92 (5.58 %)
Current smoking	508 (20.89 %)	157 (35.49 %)^1^	238 (11.42 %)^1,2^
Current alcohol use^b^	516 (21.22 %)	163 (37.35 %)^1^	471 (21.21 %)^2^
Daily exercise < 0.5 h	645 (26.61 %)	48 (14.73 %)^1^	523 (27.68 %)^2^
No. of diseases			
0	534 (21.96 %)	175 (45.44 %)^1^	435 (19.85 %)^2^
1–2	1300 (53.45 %)	221 (48.05 %)	1118 (55.43 %)
≥3	598 (24.59 %)	23 (6.51 %)	447 (24.72 %)
Daily drugs ≥ 4	663 (27.59 %)	42 (9.65 %)^1^	137 (6.92 %)^1^
GDS ≥ 8	273 (12.06 %)	5 (1.55 %)^1^	169 (8.92 %)^1,2^
MMSE < 24	249 (10.26 %)	83 (28.93 %)^1^	227 (14.28 %)^1,2^
Female	*N* = 3888	*N* = 559	*N* = 2000
Age, mean (sd)	73.85 (5.28)	73.94 (5.07)	73.73 (5.32)
Currently married	2687 (69.11 %)	398 (61.94 %)^1^	1069 (49.42 %)^1,2^
Education ≤ Middle school	2038 (52.46 %)	430 (85.35 %)^1^	1728 (87.23 %)^1^
Living alone	494 (12.71 %)	36 (7.44 %)^1^	341 (18.62 %)^1,2^
Current smoking	196 (5.04 %)	32 (5.44 %)	37 (1.91 %)^1,2^
Current alcohol use^b^	64 (1.65 %)	26 (5.42 %)^1^	51 (2.35 %)^2^
Daily exercise < 0.5 h	1074 (27.77 %)	81 (16.57 %)^1^	647 (33.26 %)^1,2^
No. of diseases			
0	661 (17.00 %)	162 (29.01 %)^1^	385 (17.85 %)^1,2^
1–2	2108 (54.22 %)	337 (60.05 %)	1167 (58.88 %)
≥ 3	1119 (28.78 %)	60 (10.94 %)	448 (23.27 %)
Daily drugs ≥ 4	1116 (29.15 %)	87 (15.31 %)^1^	127 (6.70 %)^1,2^
GDS ≥ 8	517 (14.13 %)	11 (2.87 %)^1^	203 (10.64 %)^1,2^
MMSE < 24	756 (19.47 %)	250 (54.72 %)^1^	785 (41.54 %)^1,2^

^a^percentage in Beijing rural and Hong Kong is standardized using 5-year interval of Beijing urban population

^b^current alcohol use: drink >12 alcoholic drinks in past 12 months

^1^ *p*-value < 0.05, comparing Beijing rural (2) or Hong Kong (3) with Beijing urban (1)

^2^ *p*-value < 0.05, comparing Hong Kong (3) with Beijing rural (2)

**Table 3:** Population characteristics between Beijing urban, Beijing rural and Hong Kong – age 65–74

	Mean (sd)/Freq (%)		
	Beijing urban (1)	Beijing rural (2)^a^	Hong Kong (3)^a^
Male	*N* = 1216	*N* = 333	*N* = 1372
Age, mean (sd)	70.08 (2.9)	70.17 (2.31)	69.94 (2.25)
Currently married	1112 (91.45 %)	301 (88.14 %)	1257 (91.44 %)
Education ≤ Middle school	223 (18.37 %)	174 (57.74 %)^1^	940 (69.34 %)^1,2^
Living alone	52 (4.28 %)	10 (3.44 %)	47 (3.46 %)
Current smoking	307 (25.25 %)	131 (38.87 %)^1^	172 (12.4 %)^1,2^
Current alcohol use^b^	292 (24.01 %)	134 (39.72 %)^1^	369 (26.62 %)^2^
Daily exercise < 0.5 h	294 (24.30 %)	32 (10.29 %)^1^	323 (23.47 %)^2^
No. of diseases			
0	305 (25.08 %)	135 (40.15 %)^1^	328 (23.11 %)^2^
1-2	669 (55.02 %)	184 (55.80 %)	785 (57.42 %)
≥ 3	242 (19.90 %)	14 (4.05 %)	259 (19.47 %)
Daily drugs ≥ 4	272 (22.52 %)	33 (9.93 %)^1^	95 (7.13 %)^1^
GDS ≥ 8	134 (12.04 %)	3 (0.94 %)^1^	107 (7.71 %)^1, 2^
MMSE < 24	65 (5.35 %)	49 (16.87 %)^1^	97 (7.23 %)^1, 2^
Female	*N* = 2297	*N* = 420	*N* = 1334
Age, mean (sd)	70.34 (2.75)	70.54 (2.47)	70.34 (2.56)
Currently married	1770 (77.06 %)	325 (72.10 %)^1^	874 (63.64 %)^1,2^
Education ≤ Middle school	984 (42.88 %)	296 (79.05 %)^1^	1126 (84.95 %)^1,2^
Living alone	242 (10.54 %)	25 (7.28 %)^1^	158 (12.41 %)^2^
Current smoking	120 (5.22 %)	20 (4.38 %)	24 (1.89 %)^1,2^
Current alcohol use^b^	41 (1.78 %)	16 (3.28 %)^1^	43 (3.16 %)^1^
Daily exercise < 0.5 h	568 (24.88 %)	55 (14.96 %)^1^	389 (29.21 %)^1,2^
No. of diseases			
0	438 (19.07 %)	121 (29.74 %)^1^	296 (21.09 %)^1,2^
1–2	1243 (54.11 %)	251 (57.91 %)	771 (58.3 %)
≥3	616 (26.82 %)	48 (12.34 %)	267 (20.61 %)
Daily drugs ≥ 4	617 (27.12 %)	70 (17.27 %)^1^	75 (5.91 %)^1,2^
GDS ≥ 8	291 (13.45 %)	8 (3.08 %)^1^	121 (9.44 %)^1,2^
MMSE < 24	286 (12.46 %)	155 (42.78 %)^1^	439 (34.2 %)^1,2^

^a^percentage in Beijing rural and Hong Kong is standardized using 5-year interval of Beijing urban population

^b^current alcohol use: drink >12 alcoholic drinks in past 12 months

^1^ *p*-value < 0.05, comparing Beijing rural (2) or Hong Kong (3) with Beijing urban (1)

^2^ *p*-value < 0.05, comparing Hong Kong (3) with Beijing rural (2)

**Table 4:** Population characteristics between Beijing urban, Beijing rural and Hong Kong – age 75–84

	Mean (sd)/Freq (%)		
	Beijing urban (1)	Beijing rural (2)^a^	Hong Kong (3)^a^
Male	*N* = 1102	*N* = 81	*N* = 585
Age, mean (sd)	78.34 (2.66)	78.81 (4.35)	78.18 (3.11)
Currently married	948 (86.03 %)	61 (72.05 %)^1^	474 (80.61 %)^1^
Education ≤ Middle school	366 (33.24 %)	70 (86.68 %)^1^	451 (76.49 %)^1,2^
Living alone	81 (7.35 %)	9 (11.21 %)	38 (6.8 %)
Current smoking	186 (16.88 %)	25 (33.22 %)^1^	60 (9.99 %)^1,2^
Current alcohol use^b^	209 (18.97 %)	28 (36.39 %)^1^	97 (16.24 %)^2^
Daily exercise < 0.5 h	318 (28.91 %)	16 (21.16 %)	190 (32.65 %)^2^
No. of diseases			
0	199 (18.06 %)	37 (49.30 %)^1^	98 (16.23 %)^2^
1–2	575 (52.18 %)	36 (42.26 %)	309 (53.1 %)
≥ 3	328 (29.76 %)	8 (8.44 %)	178 (30.67 %)
Daily drugs ≥ 4	358 (32.97 %)	9 (10.35 %)^1^	39 (6.64 %)^1^
GDS ≥ 8	126 (12.09 %)	2 (2.29 %)^1^	55 (9.47 %)^2^
MMSE < 24	161 (14.68 %)	32 (41.46 %)^1^	114 (19.76 %)^1,2^
Female	*N* = 1466	*N* = 136	*N* = 608
Age, mean (sd)	78.16 (2.63)	78.45 (3.31)	78.28 (2.85)
Currently married	879 (59.96 %)	73 (50.13 %)^1^	190 (30.67 %)^1,2^
Education ≤ Middle school	973 (66.37 %)	131 (96.89 %)^1^	550 (90.52 %)^1,2^
Living alone	232 (15.83 %)	11 (8.18 %)^1^	170 (28.09 %)^1,2^
Current smoking	71 (4.84 %)	12 (7.48 %)	12 (1.97 %)^1,2^
Current alcohol use^b^	22 (1.50 %)	9 (6.94 %)^1^	8 (1.28 %)^2^
Daily exercise < 0.5 h	455 (31.14 %)	26 (20.18 %)^1^	227 (37.71 %)^1,2^
No. of diseases			
0	197 (13.44 %)	40 (27.58 %)^1^	83 (13.48 %)^2^
1–2	805 (54.91 %)	84 (62.96 %)	362 (59.76 %)
≥ 3	464 (31.65 %)	12 (9.47 %)	163 (26.76 %)
Daily drugs ≥ 4	469 (32.68 %)	17 (13.25 %)^1^	46 (7.62 %)^1,2^
GDS ≥ 8	197 (14.30 %)	3 (2.76 %)^1^	71 (11.86 %)^2^
MMSE < 24	418 (28.57 %)	92 (70.59 %)^1^	311 (51.47 %)^1,2^

^a^percentage in Beijing rural and Hong Kong is standardized using 5-year interval of Beijing urban population

^b^current alcohol use: drink >12 alcoholic drinks in past 12 months

^1^ *p*-value < 0.05, comparing Beijing rural (2) or Hong Kong (3) with Beijing urban (1)

^2^ *p*-value < 0.05, comparing Hong Kong (3) with Beijing rural (2)

**Table 5:** Population characteristics between Beijing urban, Beijing rural and Hong Kong – age 85+

	Mean (sd)/Freq (%)		
	Beijing urban (1)	Beijing rural (2)^a^	Hong Kong (3)^a^
Male	*N* = 114	*N* = 5	*N* = 43
Age, mean (sd)	87.01 (2.51)	87.30 (5.38)	86.89 (3.00)
Currently married	76 (66.67 %)	3 (57.02 %)	29 (68.42 %)
Education ≤ Middle school	43 (37.72 %)	4 (85.96 %)^1^	31 (72.63 %)^1^
Living alone	16 (14.04 %)	0 (0 %)	7 (16.49 %)
Current smoking	15 (13.16 %)	1 (21.49 %)	6 (14.74 %)
Current alcohol use^b^	15 (13.16 %)	1 (21.49 %)	5 (11.58 %)
Daily exercise < 0.5 h	33 (28.95 %)	0 (0 %)	10 (24.56 %)
No. of diseases			
0	30 (26.32 %)	3 (64.47 %)	9 (20 %)
1-2	56 (49.12 %)	1 (21.49 %)	24 (56.84 %)
≥ 3	28 (24.56 %)	1 (14.04 %)	10 (23.16 %)
Daily drugs ≥ 4	33 (30.28 %)	0 (0 %)	3 (7.37 %)^1^
GDS ≥ 8	13 (12.04 %)	0 (0 %)	7 (16.49 %)
MMSE < 24	23 (20.18 %)	2 (35.53 %)	16 (36.49 %)^1^
Female	*N* = 125	*N* = 3	*N* = 58
Age, mean (sd)	87.74 (2.55)	86.50 (1.52)	87.39 (3.04)
Currently married	38 (30.40 %)	0 (0 %)	5 (7.92 %)^1^
Education ≤ Middle school	81 (65.32 %)	3 (100 %)	52 (90.5 %)^1^
Living alone	20 (16.00 %)	0 (0 %)	13 (21.49 %)
Current smoking	5 (4.00 %)	3 (100 %)^1^	1 (1.58 %)^2^
Current alcohol use^b^	1 (0.80 %)	1 (33.33 %)^1^	0 (0 %)^2^
Daily exercise < 0.5 h	51 (41.13 %)	0 (0 %)	31 (55.43 %)
No. of diseases			
0	26 (20.80 %)	1 (33.33 %)	6 (9.5 %)
1–2	60 (48.00 %)	2 (66.67 %)	34 (59.28 %)
≥ 3	39 (31.20 %)	0 (0 %)	18 (31.22 %)
Daily drugs ≥ 4	30 (25.21 %)	0 (0 %)	6 (10.41 %)^1^
GDS ≥ 8	29 (24.58 %)	0 (0 %)	11 (18.33 %)
MMSE < 24	52 (41.94 %)	3 (100 %)^1^	35 (59.95 %)^1^

^a^percentage in Beijing rural and Hong Kong is standardized using 5-year interval of Beijing urban population

^b^current alcohol use: drink >12 alcoholic drinks in past 12 months

^1^ *p*-value < 0.05, comparing Beijing rural (2) or Hong Kong (3) with Beijing urban (1)

^2^ *p*-value < 0.05, comparing Hong Kong (3) with Beijing rural (2)

The prevalence of frailty increases with age in all three cohorts, and was lower among rural compared with urban (Beijing and Hong Kong) populations (Table 6). Using the ratio of frailty index divided by life expectancy as an indicator of compression of morbidity (FI/LE), the highest ratio was observed in the Beijing urban population, followed by Hong Kong, with the Beijing rural population having the lowest ratios (Table 7). Risk factors for frailty were similar in all three populations. Those having the highest ORs were multi-morbidity (number of diseases > =3), polypharmacy (number of drugs > =4), age 85+, female gender, followed by low education level, and physical inactivity. Living alone was a risk factor for the Beijing urban and Hong Kong cohorts but not for the rural cohort. Paradoxically smoking was associated with lower risk of frailty in the two Beijing cohorts, while current alcohol use was associated with reduced risk of frailty in all three cohorts (Table 8). In the multiple logistic model, variations in ORs and their magnitude were noted between the three cohorts. The highest ORs occurred in the Beijing rural cohort, for the following risk factors: age 85+, multi-morbidity, and polypharmacy. For the Beijing urban cohort, a similar pattern was observed, although the magnitude of ORs were lower compared with the rural cohort. For Hong Kong the highest OR was multi-morbidity, being more than double the magnitude for the Beijing urban cohort. Low education level remained as significant risk factor only in the Hong Kong cohort, while current alcohol use was associated with a lower risk of frailty. For both the Beijing cohorts, these risk factors did not remain in the model; however being currently married were associated with reduced risk of frailty for both Beijing urban and rural cohorts (Table 9). The estimated attributable fraction (AF) for these risk factors are shown in Table 10. For all three cohorts, age and multi-morbidity constitute the highest attributable fraction, and were highest in the Beijing rural cohort. A major difference between the Beijing and Hong Kong cohorts is the high AF from polypharmacy in Beijing and the ‘protective’ contribution of being married; and the effect of being a teetotaler in the Hong Kong cohort.

**Table 6:** Prevalence of frailty^a^ in different areas by age and gender

	Prevalence %		
	Beijing urban (1)	Beijing rural (2)^b^	Hong Kong (3)^b^
Male			
65–74	108 (8.88 %)	4 (1.29 %)^1^	99 (7.36 %)^2^
75–84	202 (18.33 %)	6 (7.19 %)^1^	90 (15.80 %)^2^
85+	22 (19.30 %)	0 (0 %)	9 (21.40 %)
Female			
65–74	342 (14.89 %)	24 (6.59 %)^1^	223 (17.61 %)^1,2^
75–84	362 (24.69 %)	16 (12.62 %)^1^	164 (27.12 %)^2^
85+	41 (32.80 %)	1 (33.33 %)	20 (34.39 %)
Total			
65–74	450 (12.81 %)	28 (4.53 %)^1^	322 (12.91 %)^2^
75–84	564 (21.96 %)	22 (10.05 %)^1^	254 (20.94 %)^2^
85+	63 (26.36 %)	1 (13.84 %)	29 (26.69 %)

^a^Frailty index ≥ 0.25

^b^percentage in Beijing rural and Hong Kong is standardized using 5-year interval of Beijing urban population

^1^ *p*-value < 0.05, comparing Beijing rural (2) or Hong Kong (3) with Beijing urban (1)

^2^ *p*-value < 0.05, comparing Hong Kong (3) with Beijing rural (2)

**Table 7:** Mean (sd) of FI/LE ratio in different areas by age and gender

	Mean (sd) of FI/LE ratio		
	Beijing urban (1)	Beijing rural (2)^a^	Hong Kong (3)^a^
Male			
65–74	0.18 (0.11)	0.13 (0.07)^1^	0.15 (0.09)^1,2^
75–84	0.23 (0.13)	0.15 (0.16)^1^	0.19 (0.15)^1,2^
85+	0.24 (0.14)	0.17 (0.12)	0.22 (0.18)
Female			
65–74	0.21 (0.11)	0.15 (0.09)^1^	0.20 (0.10)^1,2^
75–84	0.25 (0.12)	0.19 (0.10)^1^	0.24 (0.13)^2^
85+	0.28 (0.14)	0.24 (0.12)	0.27 (0.14)
Total			
65–74	0.20 (0.11)	0.14 (0.08)^1^	0.17 (0.10)^1,2^
75–84	0.24 (0.13)	0.17 (0.14)^1^	0.21 (0.14)^1,2^
85+	0.26 (0.14)	0.20 (0.18)	0.24 (0.16)

*FI* frailty index, *LE* life expectancy

^a^FI/LE in Beijing rural and Hong Kong is standardized using 5-year interval of Beijing urban population

^1^ *p*-value < 0.05, comparing Beijing rural (2) or Hong Kong (3) with Beijing urban (1)

^2^ *p*-value < 0.05, comparing Hong Kong (3) with Beijing rural (2)

**Table 8:** Crude OR of frailty in Beijing urban, Beijing rural and Hong Kong

	Crude OR (95 % CI)		
	Beijing urban (1)	Beijing rural (2)^a^	Hong Kong (3)^a^
Female	1.50 (1.30,1.73)	2.60 (1.47,4.60)	2.07 (1.74, 2.46)^1^
Age			
65–74	Ref.	Ref.	Ref.
75–84	1.92 (1.67, 2.19)	2.35 (1.40, 3.96)	1.79 (1.50, 2.12)
85+	2.44 (1.80,3.30)	3.38 (1.17,9.77)	2.46 (1.69, 3.57)
Currently married	0.57 (0.50,0.66)	0.41 (0.25,0.67)	0.60 (0.50, 0.71)
Education ≤ Middle school	1.27 (1.11,1.45)	4.21 (1.53,11.60)^1^	1.98 (1.55, 2.53)^1^
Living alone	1.53 (1.26,1.86)	0.90 (0.34,2.41)	1.53 (1.22, 1.93)
Current smoking	0.74 (0.59,0.92)	0.22 (0.08,0.66)^1^	0.82 (0.58, 1.17)^2^
Current alcohol use^b^	0.73 (0.57,0.93)	0.35 (0.15,0.85)	0.31 (0.21, 0.45)^1^
Daily exercise < 0.5 h	1.83 (1.59,2.10)	2.96 (1.74,5.04)	1.73 (1.46, 2.05)
No. of diseases ≥ 3	7.72 (6.69,8.90)	21.81 (12.53,37.94)^1^	6.08 (5.09, 7.25)^1,2^
Daily drugs ≥ 4	5.62 (4.89,6.46)	10.62 (6.31,17.87)^1^	2.82 (2.16, 3.68)^1,2^

^a^OR in Beijing rural and Hong Kong is standardized using 5-year interval of Beijing urban population

^b^current alcohol use: drink >12 alcoholic drinks in past 12 months

^1^ *p*-value < 0.05, comparing Beijing rural (2) or Hong Kong (3) with Beijing urban (1)

^2^ *p*-value < 0.05, comparing Hong Kong (3) with Beijing rural (2)

**Table 9:** Multiple logistic regression of frailty in Beijing urban, Beijing rural and Hong Kong^a^

	Adjusted OR (95 % CI)		
	Beijing urban (1)	Beijing rural (2)^b^	Hong Kong (3)^b^
Female	1.48 (1.26,1.75)	2.97 (1.44, 6.13)	2.15 (1.76, 2.62)^1^
Age			
65–74	Ref.	Ref.	Ref.
75–84	1.71 (1.47, 2.00)	3.90 (1.97,7.73)^1^	1.59 (1.32, 1.93)^2^
85+	2.44 (1.70, 3.52)	10.13 (2.91,35.25)^1^	2.48 (1.63, 3.77)^2^
Currently married	0.70 (0.56, 0.80)	0.38 (0.20,0.73)	/
Education ≤ Middle school	/	/	1.78 (1.36, 2.33)
Current alcohol use^c^	/	/	0.54 (0.36, 0.81)
Daily exercise < 0.5 h	1.75 (1.49,2.05)	/	1.71 (1.41, 2.07)
No. of diseases ≥ 3	5.20 (4.45, 6.06)	16.31 (8.22, 32.37)^1^	6.48 (5.38, 7.81)^2^
Daily drugs ≥ 4	3.44 (2.95,4.02)	5.96 (3.06, 11.59)	/
AUC	0.819	0.908 ^1^	0.783 ^1,2^

*AUC* area under the curve

^a^multiple logistic regression with backward variable selection method

^b^OR in Beijing rural and Hong Kong is standardized using 5-year interval of Beijing urban population

^c^current alcohol use: drink >12 alcoholic drinks in past 12 months

^1^ *p*-value < 0.05, comparing Beijing rural (2) or Hong Kong (3) with Beijing urban (1)

^2^ *p*-value < 0.05, comparing Hong Kong (3) with Beijing rural (2)

**Table 10:** Attributable fraction for frailty in Beijing urban, Beijing rural and Hong Kong^a^

	Attributable fraction (%)		
	Beijing urban (1)	Beijing rural (2)^b^	Hong Kong (3)^b^
Female	32.43 %	66.33 %	53.42 %^1^
Age			
65–74	Ref.	Ref.	Ref.
75–84	41.52 %	74.36 %^1^	37.19 %^2^
85+	59.02 %	90.13 %^1^	59.64 %^2^
Currently married	−42.86 %	−163.16 %	/
Education ≤ Middle school	/	/	43.88 %
Current alcohol use^c^	/	/	−86.22 %
Daily exercise < 0.5 h	42.86 %	/	41.49 %
No. of diseases ≥ 3	80.77 %	93.87 %^1^	84.58 %^2^
Daily drugs ≥ 4	70.93 %	83.22 %	/

^a^variables from multiple logistic regression with backward variable selection method

^b^Attributable fraction in Beijing rural and Hong Kong is standardized using 5-year interval of Beijing urban population

^c^current alcohol use: drink >12 alcoholic drinks in past 12 months

^1^ *p*-value < 0.05, comparing Beijing rural (2) or Hong Kong (3) with Beijing urban (1)

^2^ *p*-value < 0.05, comparing Hong Kong (3) with Beijing rural (2)

## Discussion

The findings support the concept of FI as an indicator of biological age of based on a count of accumulated deficits, using a mathematical concept to represent aging of complex organisms, facilitating inter-population comparisons where exactly the same variables may not be available and allowing identification of factors contributing to healthy ageing or compression of morbidity. The use of FI as a public health indicator also has advantages in monitoring trends in changes of frailty with time, and also enables studies of the rate of change in frailty with time in the same cohorts. It also allows quick comparison of compression of morbidity between populations, by estimating the FI/Life expectancy ratio, which would have important implications for the planning of health and social services. This approach is fundamentally different from the use of the frailty phenotype [19] in measuring frailty in the clinical scenario, which does not include disability, as the phenotype represents a state of reduced capacity to respond to stressors that proceeds disability [4, 20].

Thus for both the Hong Kong and Beijing cohorts, FI increases with age and is higher among women, and the prevalence for urban cohorts (Hong Kong and Beijing) are similar, and higher compared with the rural cohort. However there are differences in risk factors and the attributable fraction to frailty, between the three cohorts. It is important to identify these differences as certain factor may be amenable to change. These include personal factors (socioeconomic, lifestyle) as well as differences in health and social care systems.

With respect to socioeconomic factors, the comparison shows that being married reduced the risk of frailty in both Beijing cohorts, while low education increases the risk for the Hong Kong population. The findings emphasize the importance of social support network as well as education in prevention of frailty. Although there were more current smokers in both Beijing urban and rural cohorts compared with Hong Kong, smoking was not an independent risk factor even though it is a well established risk factor for many chronic diseases, probably because multi-morbidity had been included as a model, and the survivor effect may be present. The role of alcohol may be explained by the fact that consumption is low among the older population, and may be a surrogate for social factors such as active participation in social networks and leisure activities. Being physically inactive is a risk factor in both urban, but not the rural population. This finding reaffirms the importance of physical activity in healthy aging and retarding the onset of frailty [21]. The relationship between smoking, alcohol use, physical inactivity and frailty may be bi-directional, in that frailty itself may result in avoidance of smoking, alcohol and physical inactivity.

Beijing and Hong Kong have different healthcare systems. Hong Kong government provides healthcare at low cost and is free for those who cannot afford the fees. In China, healthcare is not free at the point of access, and self payment for services is still dominant, in spite of recent introduction of medical insurance system. The items covered by insurance are limited and not all are insured. As a result the rural population may be disadvantaged. The low prevalence of multi-morbidity among the rural population may be a reflection of under reporting or under detection as a result of various financial and physical barriers to healthcare access. Under development of primary care in China results in over-reliance on hospitals, specialists, and polypharmacy, and less strong preventive health measures [1]. This may explain the higher prevalence of polypharmacy among the Beijing urban compared with the Hong Kong cohort. Polypharmacy has been shown to be one of the determinants of frailty [22]. The impact of healthcare systems on hospital admissions in relation to income and multimorbidity has been examined in detail by Wang et al. [23].

Using the FI/LE ratio as an indicator of healthy aging and a predictor of demands on health and social care systems, the higher ratio for the Beijing urban population compared with Hong Kong suggests that population ageing in China is projected to be accompanied by increasing frailty, and that there is a need to focus on measures to reduce the frailty burden. The situation for the rural population is different: they have shorter life expectancy and also lower prevalence of frailty, resulting in the lowest frailty burden. The principal of survival of the fittest likely operates in rural communities. In future urbanization of these regions may result in increasing frailty with increasing life expectancy if there are no changes to risk factor for frailty identified in this comparison.

There are limitations of this study. It is a secondary comparison of cohorts from mainland China and Hong Kong using two sets of data, rather than a simultaneous comparison at the same time point. There are many logistic problems to organizing such a large scale comparative study, and a secondary comparison with data harmonization is the most feasible option in addressing our research question. In the construction of the FI, the items included differ between Beijing and Hong Kong. Nevertheless the FI does cover a wide range of systems as recommended by Searle et al. [24]. Although more items can be included in the Hong Kong dataset, as has been previously reported (47 items) [25], we used variables common or similar to both datasets in order to compare the factors associated with frailty in both locations to address the relative contribution of associated factors and their implications for health care planning. We were not able to carry out a detailed analysis of socioeconomic factors for the same reason, although the inclusion of more socioeconomic factors into the construct of the FI would be desirable in view of the concept of frailty incorporating social vulnerability [26].

In spite of the increasing importance of frailty as a syndrome in ageing populations, the awareness and understanding of the implications for public health and primary care could be improved on, and it is hoped that this comparison will draw attention to the needs of ageing populations expressed as the concept of frailty in meeting future public health and primary care planning.

## Conclusions

This comparison draws attention to the importance of frailty prevention for ageing populations.
